# Occurrence of arbovirus infections in two riverine populations in the municipality of Humaitá, Amazonas, Brazil

**DOI:** 10.1590/0037-8682-0515-2023

**Published:** 2024-03-25

**Authors:** Jaqueline Carvalho de Oliveira Silva, Igor Rodrigo Ferreira Siqueira, Leormando Fortunato Dornelas, Cristhian Magalhães Ribeiro, João Pedro Berno Gomes, Iagor Wingenbah Guadagnin, Antonieta Relvas Pereira, Genimar Rebouças Julião, Juliana de Souza Almeida Aranha Camargo, Sergio Almeida Basano, Luís Marcelo Aranha Camargo

**Affiliations:** 1 Fundação Oswaldo Cruz, Programa de Pós-Graduação Stricto Sensu em Biologia da Interação Patógeno Hospedeiro, Manaus, AM, Brasil.; 2 Faculdade Uninassau de Vilhena, Curso de Graduação em Medicina, Vilhena, RO, Brasil.; 3 Secretaria de Estado de Saúde do Amazonas, Humaitá, AM, Brasil.; 4 Fundação Oswaldo Cruz, Departamento de Entomologia, Porto Velho, RO, Brasil.; 5 Instituto de Ciências Biomédicas V da Universidade de São Paulo, Monte Negro, RO, Brasil.; 6 Centro de Medicina Tropical de Rondônia, Porto Velho, RO, Brasil.; 7 Secretaria Municipal de Saúde de Porto Velho, Porto Velho, RO, Brasil.; 8 Secretaria Estadual de Saúde, Departamento do Centro de Pesquisa em Medicina de Rondônia, Porto Velho, RO, Brasil.; 9 Instituto Nacional De Epidemiologia Na Amazônia Ocidental, Porto Velho, RO, Brasil.

**Keywords:** Arbovirus, Riverine populations, Amazonia

## Abstract

**Background:**

The riverine communities of the Amazon comprise different social groups that inhabit the rural areas on the banks of rivers and lakes. Residents usually travel by river to rural and urban areas and are then exposed to urbanized diseases such as those caused by arbovirus infection. In Brazil, emerging diseases such as dengue, Zika, chikungunya, and those caused by infection with Oropouche and Mayaro viruses necessitate epidemiological surveillance. This study was aimed at determining the frequency of positivity for immunoglobulin (Ig)G and IgM antibodies against Zika, chikungunya, and dengue viruses and performing molecular analyses to detect viral RNA for the Zika, chikungunya, dengue virus, Oropouche, and Mayaro viruses, in the same serum samples obtained from riverside populations.

**Methods:**

This cross-sectional study was conducted in a riverside population in the Humaitá municipality of the Brazilian Amazon. More than 80% of the local population participated in this study. Entomological samples were collected to identify local mosquito vectors.

**Results:**

Analysis of 205 human serological samples revealed IgG antibodies against the dengue virus in 85 individuals. No molecular positivity was observed in human samples. Entomological analyses revealed 3,187 Diptera species, with *Mansonia* being the most frequent genus. *Aedes aegypti* and *Aedes albopictus* were not detected in the two collections.

**Conclusions:**

IgG antibodies against the dengue virus were highly prevalent, suggesting previous exposure. The absence of the arbovirus vectors *Aedes aegypti* and *Aedes albopictus* in the samples supports the hypothesis that the infections recorded likely occurred outside the riverside communities investigated.

## INTRODUCTION

The Amazon basin spans more than 7 million square kilometers, of which approximately 5 million square kilometers make up the Brazilian Amazon. It is an extensive area comprising forests and rivers and has a low demographic density, and the populations living in rural areas of the square kilometers suffer from isolation and difficulty in accessing public health services^1^ ^,^ ^2^.

The riverine communities of the Brazilian Amazon comprise different social groups, which include indigenous people, quilombolas, and migrants from other regions of the country and other countries. These populations live in rural areas along the banks of rivers and lakes of the Brazilian Amazon. Fishing, hunting, family farming, and plant and mineral extraction are their main means of income for these populations, in addition to subsidies from the federal government. They generally lack basic sanitation resources and are dependent on travel to urban areas for health care and acquisition of goods^3^ ^,^ ^4^. Travel to urban areas is usually by water and in small boats. These trips result in considerable costs for these riverine populations and are carried out only if and when there is a real need. This contact with urban areas creates opportunities for the acquisition of urbanized diseases. In the case of some diseases, however, riverine populations present with symptoms after returning to their communities, where they do not have adequate health care coverage^5^ ^,^ ^6^.

Among the main urbanized diseases in these populations are infections with arboviruses. Arboviruses are transmitted to humans by arthropods, specifically through the bite of hematophagous insects. They belong to the families *Flaviviridae*, *Togaviridae*, *Bunyaviridae*, *Rhabdoviridae*, and *Reoviridae*. There are more than 140 different arboviruses that infect humans and, of these, the most important include the dengue virus (DENV), Zika virus (ZIKV), chikungunya virus (CHIKV), yellow fever virus, West Nile virus, Mayaro virus (MAYV), and Oropouche virus (OROV). In Brazil, diseases caused by infection with the DENV, ZIKV, and CHIKV are currently considered emerging diseases^7^ ^-^ ^9^, and all three viruses are transmitted mainly by *Aedes aegypti*, which has a preference for environments close to human habitats and deposits its eggs in standing water (clean or slightly polluted). In favorable climatic conditions, mosquitoes show greater longevity, which allows females to increase their feeding and egg laying^10^ ^,^ ^11^.

 Chikungunya, dengue, and Zika rarely cause death; however, they often lead to many debilitating conditions. For example, the symptoms associated with CHIKV infection can be chronic and may last for years^12^. Owing to rapid climate change, deforestation, population migration, disorderly occupation of urban areas, globalization, international travel, and social and economic factors and the resultant lack of basic sanitation, arboviruses represent a major and constant threat to tropical populations^13^. Therefore, surveillance of arboviruses, especially in regions with difficult access to health services and quality treatment, is necessary to identify future threats to these populations, and accordingly develop prevention and health promotion strategies to meet local needs. This study was aimed at determining the frequency of positivity for immunoglobulin (Ig)G and IgM antibodies against ZIKV, CHIKV, and DENV in samples obtained from riverine populations and conducting molecular analyses for viral RNA research for ZIKV, CHIKV, DENV, OROV, and MAYV, in addition to trying to detect the presence of *Aedes aegypti* and *Aedes albopictus* in riverine regions.

## METHODS

### ● Study type

This cross-sectional study involved the analysis of serological samples for the detection of IgG and IgM antibodies against DENV, ZIKV, and CHIKV and molecular analysis for viral RNA research. Samples were collected during health care initiatives in 2019 and 2020 for routine biochemical tests in two riverine communities in the state of Amazonas. At the time, the patients signed an informed consent form authorizing the storage and use of leftover samples for future research projects. These samples are stored in the biorepository of the Institute of Biomedical Sciences of USP-5 (Rondônia) and the clinico-epidemiological data are stored in the Red Cap database. In addition, entomological samples were collected twice from these communities, during the rainy and dry seasons, to detect the presence of arbovirus vectors.

### ● Study population

The riverine communities investigated in this study are part of the municipality of Humaitá, Amazonas, and are located on the banks of the Madeira River. The two communities included the Carará community (6°44'40.6" S 62°30'16.0" W) and the Espírito Santo community (6°46'10.6" S 62°27'30.6" W), both of which are in rural areas, 7 hours by boat from the nearest city. Access to the communities is by river only. The total population of the two communities is approximately 250 individuals, and the study included children over 3 years of age, adults, and the elderly. Their data and samples were collected during local health service expeditions. Owing to the low number of residents in these communities, the sample size was chosen for convenience. Samples that did not have enough material to perform the tests used in the study or for which complete records were not available in the Red Cap database were discarded.

### ● Ethical approval

This study was conducted according to the ethical principles of human research and was approved by the research ethics committee (CAAE no. 51511415.4.0000.0013). The informed consent form was applied to both adults and minors, along with the minor assent form. Authorization for minors was granted by their legal guardians.

### ● Capture of vectors

To capture culicid eggs and larvae, oviposition traps, known as “ovitrampa” were used. This capture method was first described by Fay and Perry^14^. To capture adult specimens, different techniques were used, such as Shannon’s technique, CDC-HP light trap (close to the ground and in tree canopy), and the Nasci aspirator^15^ ^-^ ^17^. The traps were installed in the forest, in areas surrounding the forest, and in rural homes. A total of 27 ovitraps were set up for each expedition, along with 14 CDC-HP light traps, the latter being distributed in different locations throughout the expedition period. For the entomological identification of culicids, genus and species were determined using identification keys^18^ ^,^ ^19^.

### ● Laboratory tests

To detect IgG and IgM antibodies against DENV, ZIKV, and CHIKV, the TR DPP^®^ ZDC IgM/IgG immunochromatographic test (Bio-Manguinhos, Rio de Janeiro, Brazil) was used, which uses a dual resource platform. The DPP^®^ Micro Reader reads the results electronically, thus eliminating possible errors of interpretation by human reading. It also enables automatic recording of the results and computer processing of the data.

Viral RNA extractions from human serum were performed in the Laboratory of Clinical and Molecular Virology (LVCM) at Instituto de Ciências Biomédicas, Universidade de São Paulo, by using a magnetic particle processor (MagMAX™ Express, Applied Biosystems, Foster City, CA, USA) according to the manufacturer’s instructions. The extracted samples were then stored in a freezer at -80ºC until they were used for analysis. For molecular analysis of DENV, ZIKV, and CHIKV, quantitative reverse transcription polymerase chain reaction (RT-qPCR) was performed using the EM8 reagent, which is a PCR mix containing reverse transcriptase, polymerase enzymes and nucleotides and magnesium from the Allplex™ SARS-CoV-2/FluA/FluB/RSV assay kit (RV10259X; Seegene). For the PCR reaction, a concentration of 10 pM was used for each primer and probe. For molecular analysis of OROV and MAYV, conventional PCR was employed using the QuantFast SYBR Green RT-PCR kit (204156; Qiagen). The primers used are listed in Table 1.

**TABLE 1:** Primers used in RT-qPCR and conventional PCR panels for arboviruses.

Virus	Forward Primer	Reverse Primer	Probe	Reference
**DENV**	AAGGACTAGAGGTT AKAGGAGACCC	GGCCYTCTGTGCCTG GAWTGATG	AACAGCATATTGACG CTGGGARAGACC	^20^
**ZIKV**	CCGCTGCCCAACA CAAG	CCACTAACGTTCTTTT GCAGACAT	AGCCTACCTACCTTG ACAAGCAGTCAGACA CTCAA	^21^
**CHIKV**	YGAYCAYGCMGWC ACAG	AARGGYGGGTAGTCC ATGTT	CCAATGTCYTCMGCC TGGACRCCKTT	^22^
	**Primer ID**	Primer Sequence		
**OROV**	Pan Bunya Fwd Pan Bunya Ver	ATGATGTACCACAACGGAC CTAACAACACCAGCATTGA		Designed
**MAYV**	Alpha 2F Alpha 2R	GIAAYTGYAAYGTIACICARAT GCRAAIARIGCIGCIGCYTYIGGICC		^23^

**RT-qPCR:** quantitative reverse transcription polymerase chain reaction; **DENV:** dengue virus; **ZIKV:** Zika virus; **CHIKV:** chikungunya virus; **MAYV:** Mayaro virus; and **OROV:** Oropouche virus.

### ● Statistical analysis

For statistical analysis, the programs Jamovi (version 2.3.21) and Microsoft Excel (version 14.0, Office 365) were used. Clinical and epidemiological data are stored in the Red Cap database. Associations were made between these data and the results of immunochromatographic tests. The Chi-square test was used, and the Fisher test was chosen for small samples. The level of statistical significance was <5% (p < 0.05).

## RESULTS

### ● Serological analyses

Of the 212 samples available for the study, seven were excluded owing to a lack of sociodemographic data or lack of serum for performing the immunochromatographic tests. Thus, 205 patients were included in the study. Sociodemographic data are presented in Table 2.

**TABLE 2:** Sociodemographic data of the study participants.

Sociodemographic Data	N	%
**Biological sex**		
Female	113	55.2
Male	92	44.8
Total	205	100
**Age range (years)**		
3-11	64	31.3
12-29	87	42.4
30-59	45	21.9
≥60	9	4.4
Total	205	100
**Place of birth**		
Born in the community	175	85.4
Not born in the community	30	14.6
Total	205	100
**Community**		
Espírito Santo (Tabuleta)	34	16.6
Carará	171	83.4
Total	205	100

According to the Brazilian Statute of Children and Adolescents, as per Law No. 8,069, of July 13^th^, 1990, individuals up to the age of 12 years are considered children. Thus, this study included 64 children (31.3%) and 141 adults (68.7%)^24^. Most of the study participants were born and raised in the communities to which they belong (85.4%), and the rest were migrants from other cities in the state of Amazonas and neighboring states such as Rondônia and Acre.

The results in Table 3 show the socioeconomic correlations with the presence of antibodies, both IgM and IgG, against some of the viruses in this study. Indeterminate results were counted as negative in these analyses since it was not possible to confirm positivity. In the molecular analyses, none of the samples tested showed positivity for the genetic material of the viruses tested.

**TABLE 3:** Correlation of seroprevalence for the tested arboviruses and socioeconomic conditions.

Presence of antibodies against arboviruses and socioeconomic conditions					
**Place of residence**	**Positive**	%	**Negative**	**%**	**p-value**
Carará	88	80.7	83	86.5	0.272
Espírito Santo	21	19.3	13	13.5	
Total	109	100	96	100	
**Place of Birth**	**Positive**	%	**Negative**	%	**p-value**
Born in the region	94	86.2	81	84.4	0.706
Not born in the region	15	13.7	15	15.6	
Total	109	100	96	100	
**Age Range**	**Positive**	%	**Negative**	%	**p-value**
Adults	94	86.2	47	48.9	<0.001
Children	15	13.7	49	51.1	
Total	109	100	96	100	

### ● Entomological analysis

A total of 3,187 Diptera species were identified in two entomological collections, the first in February 2022 during the rainy season and the second in July 2022 during the dry season. In all, 937 specimens were collected in the first collection and 2,250 in the second; the specimens belonged to 26 species and 11 genera of Culicidae (Table 4). Most of the specimens were female (98%). More than half of the specimens (62%) were not identified at a specific level because they were damaged and, consequently, did not possess the necessary morphological characters, or belonged to complexes of cryptic species of the genus *Mansonia*, currently under debate^25^. In fact, *Mansonia* was the predominant genus in the sampling performed using the CDC-HP light traps at the bases of trees and in the active search (64.3%, n = 2,051), followed by the genera *Culex* (n = 605) and *Coquillettidia* (n = 338). Specimens of *Aedes* spp. identified were not of *Aedes aegypti* or *Aedes albopictus* and were thus classified by genus only.

**TABLE 4:** Diptera species collected in the Espírito Santo and Carará riverine communities (Amazonas state) according to sampling technique during the period from February 13^th^ to 17^th^, 2022, and July 17^th^ to 23^rd^, 2022, and their species/taxon and sex.

Community	Technique	Species	Females	Males	Total
					N %
Carará	Active search	*Coquillettidia* (*Rhynchotaenia*) *albicosta*	1		1
		*Coquillettidia* (*Rhynchotaenia*) *juxtamansonia*	1		1
		*Coquillettidia* (*Rhynchotaenia*) *venezuelensis*	10		10
		*Coquillettidia* spp.	33		33
		*Culex* spp.	13		13
		*Haemagogus* spp.	2		2
		*Mansonia* (*Mansonia*) *amazonensis*	1		1
		*Mansonia* (*Mansonia*) *humeralis*	90		90
		*Mansonia* spp.	47	2	49
		*Psorophora* spp.	23		23
		*Wyeomyia* spp.	9		9
	CDC-HP	*Aedeomyia* (*Aedeomyia*) *squamipennis*	7		7
		*Aedeomyia* spp.	1	1	2
		*Aedes* spp.	1		1
		*Anopheles* (*Nyssorhynchus*) *triannulatus*	1		1
		*Anopheles* sp.		1	1
		*Coquillettidia* (*Rhynchotaenia*) *albicosta*	4		4
		*Coquillettidia* (*Rhynchotaenia*) *chrysonotum*	1		1
		*Coquillettidia* (*Rhynchotaenia*) *hermanoi*	1		1
		*Coquillettidia* (*Rhynchotaenia*) *nigricans*	2		2
		*Coquillettidia* (*Rhynchotaenia*) *venezuelensis*	15	2	17
		*Coquillettidia* spp.	13		13
		*Culex* (*Culex*) spp.	2	1	3
		*Culex* (*Melanoconion*) *spissipes*	1		1
		*Culex* (*Melanoconion*) spp.	34	3	37
		*Culex* spp.	74	15	89
		*Haemagogus* spp.	1		1
		*Mansonia* (*Mansonia*) *amazonensis*	14		14
		*Mansonia* (*Mansonia*) *flaveola*	5		5
		*Mansonia* (*Mansonia*) *humeralis*	213	1	214
		*Mansonia* spp.	163	2	165
		*Psorophora* (*Janthinosoma*) *albipes*	1		1
		*Psorophora* spp.	9	4	13
		*Uranotaenia* (*Uranotaenia*) *cooki*	1		1
		*Uranotaenia* (*Uranotaenia*) *geometrica*		3	3
		*Uranotaenia* (*Uranotaenia*) *hystera*		1	1
		*Uranotaenia* (*Uranotaenia*) *leucoptera*	1		1
		*Uranotaenia* (*Uranotaenia*) *pulcherrima*	5		5
		*Uranotaenia* sp.		1	1
	Ovitraps	*Sabethes* (*Davismyia*) *petrocchiae*	5		5
Espírito Santo	Active search	*Aedes* spp.	1		1
		*Coquillettidia* (*Rhynchotaenia*) *venezuelensis*	4		4
		*Mansonia* (*Mansonia*) *amazonensis*	2		2
		*Mansonia* (*Mansonia*) *humeralis*	76		76
		*Mansonia* spp.	52		52
		*Wyeomyia* (*Dodecamyia*) *aphobema*	1		1
		*Wyeomyia* (*Menolepsis*) *leucostigma*	1		1
		*Wyeomyia* (*Wyeomyia*) *celaenocephala*	2		2
		*Psorophora* spp.	1		1
	CDC-HP	*Aedeomyia* (*Aedeomyia*) *squamipennis*	35		35
		*Aedeomyia* spp.	7		7
		*Aedes* spp.	1		1
		*Anopheles* (*Anopheles*) *matogrossensis*	4		4
		*Anopheles* (*Nyssorhynchus*) *oswaldoi*	3		3
		*Anopheles* (*Nyssorhynchus*) *triannulatus*	1		1
		*Anopheles* spp.	25	1	26
		*Coquillettidia* (*Rhynchotaenia*) *albicosta*	34		34
		*Coquillettidia* (*Rhynchotaenia*) *chrysonotum*	6		6
		*Coquillettidia* (*Rhynchotaenia*) *hermanoi*	2		2
		*Coquillettidia* (*Rhynchotaenia*) *juxtamansonia*	1		1
		*Coquillettidia* (*Rhynchotaenia*) *nigricans*	2		2
		*Coquillettidia* (*Rhynchotaenia*) *venezuelensis*	69		69
		*Coquillettidia* spp.	137		137
		*Culex* (*Culex*) spp.	20		20
		*Culex* (*Melanoconion*) spp.	142	1	143
		*Culex* spp.	280	19	299
		*Haemagogus* spp.	14		14
		*Mansonia* (*Mansonia*) *amazonensis*	40		40
		*Mansonia* (*Mansonia*) *flaveola*	13		13
		*Mansonia* (*Mansonia*) *humeralis*	281		281
		*Mansonia* spp.	1037	2	1,039
		*Psorophora* spp.	24		24
		*Uranotaenia* (*Uranotaenia*) *geometrica*	2	1	3
		*Uranotaenia* (*Uranotaenia*) *nataliae*	1		1
		**Total**	**3,126**	**61**	**3,187**

In the entomological collection techniques used in the study, which included the active search of breeding sites, ovitraps and CDC-HP light traps were placed at tree bases. The ones with the highest productivity in terms of the number of specimens caught were the CDC-HP light traps placed at tree bases; however, some specimens of typically wild genera, such as *Sabethes* and *Wyeomyia*, were collected only by the active searching of breeding sites and by using ovitraps.

## DISCUSSION

This study showed a high seroprevalence for the arboviruses tested, which demonstrates that the study population has contact with diseases caused by arbovirus infection and is susceptible to the development of problems that these viruses can cause. Studies focused on rural populations of the Amazon are rare, and we believe that this is the first study on the seroprevalence of arboviruses in riverine populations of the Amazon to date. Studies in Malaysia have shown that arboviruses, especially the DENV, are no longer confined to urban areas because similar rates of seroprevalence have been recorded in urban and rural areas^26^ ^,^ ^27^.

The most frequent seropositivity in our study was for the DENV, corroborating the findings of the study by Júnior et al.^28^, which states that dengue is a neglected tropical disease whose burden has increased the most in recent decades in Brazil. The authors estimated that in 2016, dengue accounted for more than 92,000 disability-adjusted life years (DALYs) in the country, with a rate of 44.87 DALYs per 100,000 inhabitants.

In Brazil, the dengue surveillance system is based on passive notification of health care centers via laboratory diagnosis. This surveillance method may underestimate the incidence of dengue owing to underreporting of symptomatic and asymptomatic cases^29^. Between the years 2018 and 2021, 449 cases of dengue were registered in the municipality of Humaitá, which is the closest urban health care support center for the study population. Interestingly, in 2020, no cases were registered, which is most likely related to the coronavirus disease 2019 pandemic^30^. However, according to DATASUS^30^, in the period from 2018 to 2021, cases of infection were more frequent in the age group of 15 to 59 years. This corresponds to 373 cases of the 449 registered (83.07%), similar to the number of adults infected by any one of the arboviruses in this study (86.26%).

Martins et al*.* ^31^ conducted a study of the seroprevalence of arboviruses in Amazonian children in Acre and found that most children with detectable antibodies against the DENV were not clinically diagnosed by a health professional and had symptoms detected by their family and that, in fact, the number of children who with dengue may be up to 10 times higher than the numbers registered in the digital health system. This fact is justified by the difficulty of clinically diagnosing arboviral infections in children, since this age group presents with several febrile diseases and no specific symptoms, often because children do not know how to report them. This further increases the importance of serological diagnosis and the study of seroprevalence in populations to determine the true picture of infections^29^.

Although many studies affirm that women are at a higher risk for arbovirus infection, since they spend longer periods at home, considering the domestic habits of the *Aedes aegypti* vector^32^ ^,^ ^33^, this study demonstrated a higher prevalence of previous infections with the DENV in men (p < 0.018) (Table 5). This can be justified by the fact that infections did not occur in rural households but in urban areas, with trips to the local towns being mostly undertaken by men and, for cultural reasons, women remaining at home to perform chores and take care of children. One study conducted in a settlement in the rural area of the state of Acre in the Brazilian Amazon, also demonstrated a higher prevalence of arboviral infections in men. This was justified by their migratory activities to urban areas, which corroborates the results of our study^34^.

**TABLE 5:** Presence of IgM and IgG antibodies against dengue, Zika, and chikungunya viruses in the study population according to the biological sex.

Presence of antibodies against dengue, Zika, and chikungunya viruses							
**Dengue IgM**							
**Biological sex**	**Positive**	%	**Negative**	**%**	**Ind.**	**%**	**p-value**
Female	3	100	107	54.04	3	75	0.204
Male	0	0	91	45.96	1	25	
Total	3	100	198	100	4	100	
Dengue IgG							
**Biological sex**	**Positive**	%	**Negative**	**%**	**Ind.**	**%**	**p-value**
Female	41	48.24	69	63.30	3	27.27	0.018
Male	44	51.76	40	36.70	8	72.73	
Total	85	100	109	100	11	100	
**Zika IgM**							
**Biological sex**	**Positive**	**%**	**Negative**	**%**	**Ind.**	**%**	**p-value**
Female	0	0	113	55.39	-	-	0.506
Male	1	100	91	44.61	-	-	
Total	1	100	204	100	-	-	
Zika IgG							
**Biological sex**	**Positive**	%	**Negative**	**%**	**Ind.**	%	**p-value**
Female	3	50	110	56.12	0	0	0.148
Male	3	50	86	43.88	3	100	
Total	6	100	196	100	3	100	
Chikungunya IgM							
**Biological sex**	**Positive**	**%**	**Negative**	**%**	**Ind.**	**%**	**p-value**
Female	10	62.5	102	54.54	1	50	0.819
Male	6	37.5	85	45.46	1	50	
Total	16	100	187	100	2	100	
Chikungunya IgG							
**Biological sex**	**Positive**	**%**	**Negative**	**%**	**Ind.**	**%**	**p-value**
Female	26	59.52	86	54.43	1	20%	0.192
Male	16	38.09	72	45.57	4	80%	
Total	42	100	158	100	5	100%	

On comparing infections with any type of arbovirus among various age groups, via statistical analyses, we found a higher prevalence of infection among adults. This finding reinforces the hypothesis that infections occur in urban areas, and because children travel less to cities owing to travel costs, there is lower exposure and lower seroprevalence. It is important to emphasize that most of the patients in the study were born in the communities investigated (85%); therefore, they probably acquired these arboviral infections when visiting urban areas.

Infection by the CHIKV was more significant compared with ZIKV infections in this study population. Research has shown that infections with CHIKV resulted in decompensation associated with pre-existing diseases, such as heart, kidney, and liver diseases; diabetes; hypertension; and systemic lupus erythematosus. Furthermore, the need for hospitalization owing to CHIKV infection is related to the exacerbation of pre-existing chronic non-communicable diseases (NCDs)^35^. In 2022, Pereira et al.^36^ conducted a study of chronic NCDs in the same population as the present study and found a high prevalence of these diseases, which resulted in an even greater alert regarding the exposure of this population to this virus. 

Although infections with MAYV and OROV are highly frequent infections in the Amazon region^37^ ^-^ ^40^, we did not find positivity of these viruses in molecular tests during the research of the genetic material of these viruses in the samples tested. Owing to limitations of the tests to identify antibodies against these viruses, only molecular analyses were performed.

Sanitary vigilance is important in riverine populations because, when individuals from these populations go into the forest for labor and cultural activities, they may get exposed to wild viral species. Other factors, such as intensification of agriculture, livestock farming, mining, and the installation of large hydroelectric dams, are known to increase deforestation and are associated with human mobility and density. These factors change the ecological patterns of virus-vector-host interactions and result in a scenario that is totally favorable to the spread of diseases such as dengue, Zika, and chikungunya^41^ ^,^ ^42^. 

No *Aedes aegypti* and *Aedes albopictus* specimens were identified in the entomological collections in this study. This is in contrast with the study of Sacramento et al.^43^, in which they performed seroprevalence tests for arboviruses and entomological surveillance in a village in the state of Ceará and identified the presence of mosquitoes in all the houses of the village under study. This fact reinforces the hypothesis that the infections recorded in our study occurred outside the riverine communities, more specifically in urban areas, or that another vector assumed the role of vectorization for these diseases. Molecular studies of the captured mosquitoes are underway (FIOCRUZ - RO) to evaluate the possibility of potential vectors for these arboviruses, since several studies have proven the capacity of other vectors for the transmission of DENV, ZIKV, and CHIKV^44^ ^-^ ^48^. 

Although the tests used in this study have high sensitivity and specificity compared with those of other methods such as enzyme-linked immunosorbent assay, one of the limitations of the present study is that it was not possible to perform an assay to distinguish between the different dengue serotypes and confirm or rule out cross-reactivity. Another limitation is that sampling was performed by convenience (non-randomized), although it did account for 85% of the estimated population.

In Brazil, the arbovirus control model remains traditionally limited to specific investments in campaigns against mosquitoes and guidance through the media, with little effective impact on the fight against these diseases in general. There is a lack of innovation to address these endemic diseases, and there is a need for improvement in the training of health professionals, elimination of breeding sites, and health education initiatives for the general population. A study conducted in Icaraí-Caucaia (state of Ceará) on health education for dengue prevention and control concluded that health education initiatives were conducted ineffectively, without dialogue between health professionals and the general public^49^. Therefore, knowledge about these arboviruses and the ways to manage infections with them must be spread to remote communities, which do not know much about these pathogens, so that the population knows how to recognize the signs and symptoms of any complications that may occur and seek appropriate medical assistance.

The data presented suggest it is possible to highlight the importance of health education in this population and community health workers to enable them to manage infections with arboviruses and recognize symptoms. When communities get sick, diseases may aggravate, and at present, there is no medical assistance available for the necessary interventions in the short (severe dengue), medium (prenatal ZIKV infection) and long term (CHIKV-related arthrosis).

## References

[1] Ellwanger JH, Kulmann-Leal B, Kaminski VL, Valverde-Villegas JM, Veiga ABGD, Spilki FR (2020). Beyond diversity loss and climate change: Impacts of Amazon deforestation on infectious diseases and public health. An Acad Bras Cienc.

[2] Gama ASM, Secoli SR (2020). Self-medication practices in riverside communities in the Brazilian Amazon Rainforest. Rev Bras Enferm.

[3] Gama ASM, Fernandes TG, Parente RCP, Secoli SR (2018). Inquérito de saúde em comunidades ribeirinhas do Amazonas, Brasil. Cad Saúde Pública.

[4] Guimarães AF, Barbosa VLM, Silva MP da, Portugal JKA, Reis MH da S, Gama ASM (2020). Access to health services for riverside residents in a municipality in Amazonas State, Brazil. Rev Panamazonica Saude.

[5] Fonseca ET da, Castro DSG, Morais RC, Bandeira FJS (2023). Challenges of health care in riverside populations.

[6] Garnelo L, Parente RCP, Puchiarelli MLR, Correia PC, Torres MV, Herkrath FJ (2020). Barriers to access and organization of primary health care services for rural riverside populations in the Amazon. Int J Equity Health.

[7] Ciota AT (2019). The role of co-infection and swarm dynamics in arbovirus transmission. Virus Res.

[8] Wu P, Yu X, Wang P, Cheng G (2019). Arbovirus lifecycle in mosquito: acquisition, propagation and transmission. Expert Rev Mol Med.

[9] Young PR (2018). Arboviruses: A Family on the Move. Adv Exp Med Biol.

[10] Rocha Taranto MF, Pessanha JEM, dos Santos M, dos Santos Pereira Andrade AC, Camargos VN, Alves SN (2015). Dengue outbreaks in Divinopolis, south-eastern Brazil and the geographic and climatic distribution of Aedes albopictus and Aedes aegypti in 2011-2012. Trop Med Int Health.

[11] Donalisio MR, Freitas ARR, Zuben APBV (2017). Arboviruses emerging in Brazil: challenges for clinic and implications for public health. Rev Saude Publica.

[12] Huang YJS, Higgs S, Vanlandingham DL (2019). Emergence and re-emergence of mosquito-borne arboviruses. Curr Opin Virol.

[13] Lima-Camara TN (2016). Emerging arboviruses and public health challenges in Brazil. Rev Saude Publica.

[14] Fay RW, Perry AS (1965). Laboratory Studies of Oviposilional Preferences of Aedes aegypti. Mosq News.

[15] Nasci RS (1981). A lightweight battery-powered aspirator for collecting resting mosquitoes in the field. Mosq News.

[16] Sudia WD, Chamberlain RW (1988). Battery-operated light trap, an improved model. By W. D. Sudia and R. W. Chamberlain, 1962. J Am Mosq Control Assoc.

[17] Shannon RC (1939). Methods for Collecting and Feeding Mosquitoes in Jungle Yellow Fever Studies 1. Am J Trop Med Hyg.

[18] Consoli RAGB, Oliveira RL de (1994). Principais mosquitos de importância sanitária no Brasil.

[19] Forattini OP (2002). Culicidologia médica.

[20] Wahed AAE, Patel P, Faye O, Thaloengsok S, Heidenreich D, Matangkasombut P (2015). Recombinase Polymerase Amplification Assay for Rapid Diagnostics of Dengue Infection. PLoS One.

[21] Lanciotti RS, Kosoy OL, Laven JJ, Velez JO, Lambert AJ, Johnson AJ (2008). Genetic and serologic properties of Zika virus associated with an epidemic, Yap State, Micronesia, 2007. Emerg Infect Dis.

[22] Lopez-Jimena B, Wehner S, Harold G, Bakheit M, Frischmann S, Bekaert M (2018). Development of a single-tube one-step RT-LAMP assay to detect the Chikungunya virus genome. PLoS Negl Trop Dis.

[23] Sánchez-Seco MP, Rosario D, Quiroz E, Guzmán G, Tenorio A (2001). A generic nested-RT-PCR followed by sequencing for detection and identification of members of the alphavirus genus. J Virol Methods.

[24] Brasil (1990). Lei n^o^ 8.069, de 13 de julho de 1990. O Estatuto Criança e Adolescente.

[25] Scarpassa VM, Batista ET, Ferreira V da C, Santos VA dos, Roque RA, Tadei WP (2022). DNA barcoding suggests new species for the Mansonia subgenus (Mansonia, Mansoniini, Culicidae, Diptera) in the area surrounding the Jirau hydroelectric dam, Porto Velho municipality, Rondônia state, Brazil. Acta Trop.

[26] Chew CH, Woon YL, Amin F, Adnan TH, Abdul Wahab AH, Ahmad ZE (2016). Rural-urban comparisons of dengue seroprevalence in Malaysia. BMC Public Health.

[27] Muhammad Azami NA, Salleh SA, Neoh HM, Syed Zakaria SZ, Jamal R (2011). Dengue epidemic in Malaysia: Not a predominantly urban disease anymore. BMC Res Notes.

[28] Massad E, Lobao-Neto A, Kastner R, Oliver L, Gallagher E (2022). Epidemiology and costs of dengue in Brazil: a systematic literature review. Int J Infect Dis.

[29] Coelho I, Haguinet F, JK B. Colares, Z C. B. Coelho, F M. C. Araújo, Dias Schwarcz W (2020). Dengue Infection in Children in Fortaleza, Brazil: A 3-Year School-Based Prospective Cohort Study. Am J Trop Med Hyg.

[30] Brasil, Ministério da Saúde. Departamento de Informática do SUS (DATASUS) (2023). DENGUE - Notificações registradas no Sistema de Informação de Agravos de Notificação - Amazonas.

[31] Martins AC, Pereira TM, Oliart-Guzmán H, Delfino BM, Mantovani SAS, Braña AM (2014). Seroprevalence and Seroconversion of Dengue and Implications for Clinical Diagnosis in Amazonian Children. Interdiscip Perspect Infect Dis.

[32] Carrillo MA, Cardenas R, Yañez J, Petzold M, Kroeger A (2023). Risk of dengue, Zika, and chikungunya transmission in the metropolitan area of Cucuta, Colombia: cross-sectional analysis, baseline for a cluster-randomised controlled trial of a novel vector tool for water containers. BMC Public Health.

[33] Wenham C, Nunes J, Matta GC, Nogueira C de O, Valente PA, Pimenta DN (2020). Gender mainstreaming as a pathway for sustainable arbovirus control in Latin America. PLoS Negl Trop Dis.

[34] Silva-Nunes M da, Souza VAF de, Pannuti CS, Sperança MA, Terzian ACB, Nogueira ML (2008). Risk Factors for Dengue Virus Infection in Rural Amazonia: Population-based Cross-sectional Surveys. Am J Trop Med Hyg.

[35] Cunha RV, Trinta KS, Montalbano CA, Sucupira MVF, de Lima MM, Marques E (2017). Seroprevalence of Chikungunya Virus in a Rural Community in Brazil. PLoS Negl Trop Dis.

[36] Pereira AR, Camargo JS de AA, Basano S de A, Camargo LMA (2022). Prevalence of chronic noncommunicable diseases and their associated factors in adults over 39 years in riverside population in the Western Brazilian Amazon region. J Hum Growth Dev.

[37] Caicedo EY, Charniga K, Rueda A, Dorigatti I, Mendez Y, Hamlet A (2021). The epidemiology of Mayaro virus in the Americas: A systematic review and key parameter estimates for outbreak modelling. PLoS Negl Trop Dis.

[38] do Nascimento VA, Santos JHA, Monteiro DC da S, Pessoa KP, Cardoso AJL, de Souza VC (2020). Oropouche virus detection in saliva and urine. Mem Inst Oswaldo Cruz.

[39] Pereira-Silva JW, Ríos-Velásquez CM, de Lima GR, Marialva dos Santos EF, Belchior HCM, Luz SLB (2021). Distribution and diversity of mosquitoes and Oropouche-like virus infection rates in an Amazonian rural settlement. PLoS One.

[40] Queiroz JA da S, Botelho-Souza LF, Nogueira-Lima FS, Rampazzo R de CP, Krieger MA, Zambenedetti MR (2020). Phylogenetic Characterization of Arboviruses in Patients Suffering from Acute Fever in Rondônia, Brazil. Viruses.

[41] Batista PM, Andreotti R, Chiang JO, Ferreira MS, Vasconcelos PF da C (2012). Seroepidemiological monitoring in sentinel animals and vectors as part of arbovirus surveillance in the state of Mato Grosso do Sul, Brazil. Rev Soc Bras Med Trop.

[42] Gomes H, de Jesus AG, Quaresma JAS (2023). Identification of risk areas for arboviruses transmitted by Aedes aegypti in northern Brazil: A One Health analysis. One Health.

[43] Sacramento RHM, de Carvalho Araújo FM, Lima DM, Alencar CCH, Martins VEP, Araújo LV (2018). Dengue Fever and Aedes aegypti in indigenous Brazilians: seroprevalence, risk factors, knowledge and practices. Trop Med Int Health.

[44] Guedes DR, Paiva MH, Donato MM, Barbosa PP, Krokovsky L, Rocha SW dos S (2017). Zika virus replication in the mosquito Culex quinquefasciatus in Brazil. Emerg Microbes Infect.

[45] Guo X xia, Li C xiao, Deng Y qiang, Xing D, Liu Q mei, Wu Q (2016). Culex pipiens quinquefasciatus: a potential vector to transmit Zika virus. Emerg Microbes Infect.

[46] Lourenço-de-Oliveira R, Failloux AB (2017). High risk for chikungunya virus to initiate an enzootic sylvatic cycle in the tropical Americas. PLoS Negl Trop Dis.

[47] O’Donnell KL, Bixby MA, Morin KJ, Bradley DS, Vaughan JA (2017). Potential of a Northern Population of Aedes vexans (Diptera: Culicidae) to Transmit Zika Virus. J Med Entomol.

[48] Serra OP, Cardoso BF, Ribeiro ALM, dos Santos FAL, Slhessarenko RD (2016). Mayaro virus and dengue virus 1 and 4 natural infection in culicids from Cuiabá, state of Mato Grosso, Brazil. Mem Inst Oswaldo Cruz.

[49] Ferreira DT de O, Atanaka M, Martinez Espinosa M, Schuler-Faccini L, da Silva Caldeira A, da Silva JH (2021). Recent dengue virus infection: epidemiological survey on risk factors associated with infection in a medium-sized city in Mato Grosso. Sao Paulo Med J.

